# First-line chemotherapy with selective internal radiation therapy for intrahepatic cholangiocarcinoma: The French ACABi GERCOR PRONOBIL cohort

**DOI:** 10.1016/j.jhepr.2024.101279

**Published:** 2024-11-20

**Authors:** Nicolas Adamus, Julien Edeline, Julie Henriques, Nadim Fares, Thierry Lecomte, Anthony Turpin, Dewi Vernerey, Mathilde Vincens, Brice Chanez, David Tougeron, Christophe Tournigand, Eric Assenat, Matthieu Delaye, Sylvain Manfredi, Olivier Bouché, Nicolas Williet, Angelique Vienot, Lorraine Blaise, Léo Mas, Cindy Neuzillet, Alice Boilève, Gaël S. Roth

**Affiliations:** 1 Univ. Grenoble Alpes, Department of Hepato-Gastroenterology and Digestive Oncology, CHU Grenoble Alpes, Grenoble; Association pour l'étude des Cancers et Affections des voies Biliaires (ACABi); GERCOR, Paris, France; 2 Department of Medical Oncology, Centre Eugène Marquis, Rennes, France and INSERM, Univ Rennes, COSS (Chemistry Oncogenesis Stress Signaling), UMR_S 1242, Rennes, France; 3 Department of Methodology and Quality of Life Unit in Oncology, University Hospital of Besançon; University Bourgogne Franche-Comté, EFS, INSERM, UMR RIGHT, Besançon, France; 4 Department of Digestive Oncology, CHU of Toulouse, Rangueil Hospital, Toulouse, France; 5 Department of Hepato-Gastroenterology and Digestive Oncology, CHU Tours and UMR INSERM U 1069, Trousseau Hospital, Tours University, Tours, France; 6 Department of Medical Oncology, CHU Lille, CNRS UMR9020, INSERM UMR-S 1277-Canther-Cancer Heterogeneity, Plasticity and Resistance to Therapies, Lille University; GERCOR, Paris, France; 7 Department of Medical Oncology and Hepato-Gastroenterology, Hospices Civils de Lyon, Lyon, France; 8 Department of Medical Oncology, Paoli-Calmette Institute, Marseille, France; 9 Department of Hepato-Gastroenterology, Poitiers University Hospital, Poitiers, France; 10 Department of Medical Oncology, Henri Mondor Hospital, AP-HP, Paris-East Créteil University and INSERM, IMRB, Creteil, France; 11 Department of Medical Oncology, CHU St Eloi, Montpellier University 2, CNRS, UMR 5535, Institute of Molecular Genetic, Montpellier 1 University, Montpellier, France; 12 GI Oncology, Department of Medical Oncology, Institute Curie - Site Saint Cloud, Versailles Saint-Quentin University, Paris Saclay University, Saint-Cloud; Molecular Oncology, UMR144, Institute Curie, Paris, France; 13 Bourgogne University, CHU Dijon-Bourgogne, INSERM U1231. BP 87 900, Dijon, France; 14 Department of Gastroenterology and Digestive Oncology, CHU Reims, Université Reims Champagne Ardennes (URCA), Reims, France; 15 Department of Hepato-Gastroenterology and Gastrointestinal Oncology, University Institute of Cancerology and Hematology of Saint-Etienne (ICHUSE), Saint-Etienne, France; 16 Department of Medical Oncology, University Hospital of Besançon, Besançon, France; 17 Liver unit, Avicenne Hospital, Universitaires Paris-Seine-Saint-Denis Hospital, Assistance-Publique Hôpitaux de Paris, Bobigny; Unité de Formation et de Recherche Santé Médecine et Biologie Humaine, Université Paris 13, Communauté d'Universités et Etablissements Sorbonne Paris Cité; Centre de Recherche des Cordeliers, Sorbonne Université, Inserm, Université de Paris, team « Functional Genomics of Solid Tumors », Paris, France; 18 Department of Oncology, Pitié-Salpêtrière Hospital, AP-HP; University of La Sorbonne, Paris, France; 19 Department of Medicine, Gustave Roussy Hospital, INSERM U1279, Villejuif; University of Paris Saclay, Orsay, France; 20 Univ. Grenoble Alpes/Department of Hepato-Gastroenterology and Digestive Oncology, CHU Grenoble Alpes/Institute for Advanced Biosciences, CNRS UMR 5309-INSERM U1209, Grenoble, France

**Keywords:** cholangiocarcinoma, biliary tract cancer, SIRT, radioembolization, CISGEM, GEMOX

## Abstract

**Background & Aims:**

Selective internal radiation therapy (SIRT) is a promising option for liver-only unresectable intrahepatic cholangiocarcinoma (iCCA). The Real-SIRTCCA study retrospectively assessed the benefit of adding SIRT to chemotherapy in this setting within the French nationwide observational cohort ACABi-GERCOR-PRONOBIL.

**Methods:**

Inclusion criteria were advanced iCCA with limited or no extrahepatic disease, treated with first-line gemcitabine plus platinum chemotherapy +/- concurrent SIRT. All patients treated with chemotherapy and concurrent SIRT were included. To ensure groups’ similarity, a rigorous selection was applied to the chemo-only group, with exclusion of patients with liver involvement >50% and extrahepatic metastases. The primary outcome was progression-free survival (PFS). Secondary outcomes were overall survival (OS), objective response rate (ORR) and tumor resection rate. Propensity score and inverse probability of treatment weighting (IPTW) propensity approaches were used to address confounding factors between groups.

**Results:**

Between July 2007 and December 2023, 277 patients met the Real-SIRTCCA inclusion criteria, with 88 in the chemo-SIRT group and 189 in chemo-only group. Chemo-SIRT was associated with longer PFS (median = 10.8 *vs.* 5.5 months, hazard ratio [HR] 0.54, 95% CI 0.41-0.71, *p <*0.0001), a trend for longer OS (median = 22.5 *vs.* 15.1 months, HR 0.76, 95% CI 0.57-1.01), higher ORR (58.3% *vs.* 28.5%, odds ratio [OR] 3.51, 95% CI 2.03-6.09, *p <*0.0001), and resection rate (18.7% *vs*. 8.8%, *p* = 0.0279) compared to chemo-alone. After IPTW, the superiority of chemo-SIRT was confirmed with better PFS (HR 0.55, 95% CI 0.45-0.66, *p <*0,0001), OS (HR 0.70, 95% CI 0.58-0.85, *p* = 0.0004), ORR (OR 3.17, 95% CI 2.18-4.49, *p <*0.0001) and resection rate (OR 2.94, 95% CI 1.71-5.03, *p <*0.0001).

**Conclusions:**

Adding SIRT to first-line chemotherapy significantly improved survival outcomes, ORR, and secondary tumor resection rates in locally advanced iCCA. Prospective randomized data are needed to confirm these results.

**Impact and implications:**

Herein, we report the results of the Real-SIRTCCA study, comparing the efficacy of the gemcitabine-platinum systemic first-line chemotherapy with or without selective internal radiation therapy (SIRT) in 277 patients with locally advanced intrahepatic cholangiocarcinoma within the cohort ACABi-PRONOBIL. An improvement of progression-free survival, overall survival, tumor response and secondary surgical resection rate was observed in favor of chemo-SIRT, before adjustment and after inverse probability of treatment weighting propensity score analyses. Even though prospective randomized data would be needed to confirm these findings, we believe that this study constitutes new evidence of the potential benefit of combining SIRT with chemotherapy. The safety and efficacy of this strategy whether as a bridge to intent-to-cure strategies or in a palliative setting, should encourage its adoption in a larger panel of clinical centers, or at very least, prompt clinicians to refer their patients to centers where SIRT is performed.

**Clinical trial number:**

NCT04935853.

## Highlights

• SIRT is a local treatment option for unresectable locally advanced iCCA.
• Real-SIRTCCA retrospectively compares chemotherapy +/- SIRT in iCCA within the ACABi/PRONOBIL cohort.
• In Real-SIRTCCA, SIRT leads to better outcomes with longer PFS and OS.
• SIRT intensification improves objective response rate and rates of secondary tumor resection.
• Adding SIRT to first-line chemotherapy is feasible for locally advanced iCCA.

## Graphical abstract

**Figure:**
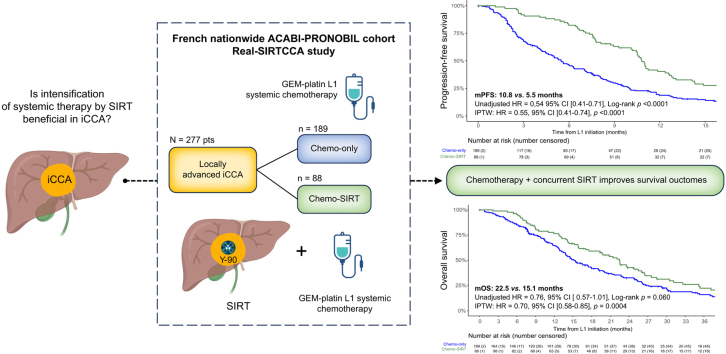

## Introduction

Intrahepatic cholangiocarcinoma (iCCA) is the second most common malignant primary liver cancer, accounting for 10-15% of cases.[1], [2], [3] Its incidence is rising worldwide, particularly in Western countries, due to the increasing prevalence of chronic liver diseases. Surgery remains the only curative treatment available. However, most patients are diagnosed at a locally advanced or metastatic stage, with only 11-22% of patients with iCCA being eligible for surgery.[4], [5], [6]

In the unresectable setting, the standard first-line systemic therapy is based on the combination of gemcitabine and cisplatin (CISGEM) since 2010,^7^ with the recent addition of programmed cell death (PD)/PD-ligand(L)-1 checkpoint inhibitors such as durvalumab or pembrolizumab.^8^^,^^9^ In case of liver-only advanced iCCA, tumor burden is a determinant of patient survival due to the risk of tumor-related liver failure and biliary complications.^10^ However, despite these treatment options, the efficacy of systemic therapy remains limited, with CISGEM and CISGEM-durvalumab achieving objective response rates (ORR) of 18.7 and 26.7% and with median progression-free survival (PFS) of 5.7 and 7.2 months, respectively, according to the TOPAZ-1 phase III trial.^8^

Liver-directed locoregional treatment might be considered to reduce tumor volume, either to enable surgical resection or to preserve liver function and improve prognosis. The role of locoregional treatments such as selective internal radiation therapy (SIRT) using Yttrium-90 microspheres or transarterial chemoembolization, in the management of cholangiocarcinoma is still debated due to the lack of prospective randomized data.[11], [12], [13], [14] Recently, SIRT combined with CISGEM has shown promising results in the MISPHEC single-arm phase II trial with median PFS of 14 months, overall survival (OS) of 22 months, and ORR of 40%.^15^ Since 2018, SIRT using yttrium-90 microspheres has been reimbursed in France in the first-line setting, in combination with standard systemic chemotherapy for patients with unresectable or recurrent iCCA, no extrahepatic disease, a tumor burden <50%, and preserved performance status and liver function. International guidelines endorse the combination chemotherapy with SIRT as a therapeutic option in this context,^11^^,^^12^ and this strategy is increasingly being utilized. However, access to SIRT remains uneven across the country and its benefit is still questionable.

The aim of the Real-SIRTCCA study is to retrospectively compare the efficacy of the gemcitabine-platinum systemic first-line chemotherapy and SIRT (chemo-SIRT) combination *vs.* chemotherapy alone (chemo-only) in patients with iCCA, with liver-only or with limited extrahepatic tumor burden, within the French nationwide ACABi-PRONOBIL observational cohort.

## Patients and methods

### Patient selection

ACABi-PRONOBIL is a French retro-prospective cohort sponsored by GERCOR and labelled by the French intergroup PRODIGE (Fédération Francophone de Cancérologie Digestive, UNICANCER, GERCOR). It includes patients aged 18 years or older with histologically or cytologically confirmed biliary tract cancer, diagnosed between 2003 and 2024.

Real-SIRTCCA is an ancillary study, including all consecutive patients from ACABi-PRONOBIL with advanced iCCA with limited or no extrahepatic tumor burden. Included patients were treated in a routine setting with first-line gemcitabine-platin-based chemotherapy (gemcitabine-oxaliplatin [GEMOX],^16^ CISGEM,^17^ CISGEM-durvalumab^8^) with or without concurrent SIRT. In the chemo-SIRT group, all patients meeting the inclusion criteria and treated with chemotherapy and concurrent SIRT were included. Concurrent SIRT was defined as a procedure performed up to 2 months before starting chemotherapy, at the same time, or within a maximum of 2 months post-chemotherapy. Patients with SIRT performed between 2 and 6 months after chemotherapy were excluded to minimize confounding bias while cases with SIRT beyond 6 months after L1 could be included in the chemo-only group, as this might not represent an intent-to offer SIRT as first-line treatment.

To limit bias from selecting patients with poorer prognosis, a more rigorous selection process was applied to the control group to ensure the inclusion of patients who would be theoretically good candidates for SIRT. Thus, in the chemo-only group, patients with an Eastern Cooperative Oncology Group (ECOG) performance status greater than 1, liver involvement exceeding 50%, or the presence of extrahepatic metastatic spread beyond distant lymph nodes were excluded. In contrast, these criteria did not exclude patients in the chemo-SIRT group, as the study aimed to evaluate the combination chemotherapy and SIRT in a real-life setting. Intra- and extrahepatic spread work-up comprised a contrast-enhanced thoraco-abdomino-pelvic CT scan with or without additional liver MRI and/or a PET scan.

The study was conducted in accordance with Good Clinical Practice and the Declaration of Helsinki^18^ and was registered on ClinicalTrial.gov (NCT04935853).

### Outcomes

The primary objective was to compare PFS between chemotherapy with SIRT and chemo-alone in the real-life setting. PFS was defined as the time between the initiation of first-line treatment (chemotherapy or SIRT, whichever was initiated first) and the date of disease progression or death, whichever occurred first. Patients alive without progression were censored at the date of their last follow-up. Secondary outcomes included OS, defined as the time between the initiation of first-line treatment and the date of death, the ORR, defined as the proportion of patients with complete or partial response as their best response during treatment according to RECIST 1.1 criteria, the secondary surgical resection rate, defined as the rate of surgeries performed after the initiation of first-line treatment, and safety, assessed according to NCI CTCAE v 5.0 criteria.

### Statistical analysis

Patient characteristics were described for the overall population with medians and interquartile ranges (Q1-Q3) for continuous variables, and frequencies and percentages for categorical variables. Baseline characteristics were compared between the chemo-SIRT and chemo-only groups with the Wilcoxon test for continuous variables and the chi-square test or Fisher’s exact test for categorical variables. Follow-up was estimated with the reverse Kaplan-Meier method. PFS and OS were estimated with the Kaplan-Meier method, described with medians and 95% CIs. Association between groups and survivals was estimated with univariable Cox proportional hazards regression models, providing hazard ratios (HRs) with 95% CIs. The proportionality assumption in Cox regression models was tested using Schoenfeld residuals. For analyses where this assumption was violated, the difference in restricted mean survival between the treatment groups was estimated. Sensitivity analyses of PFS and OS were run in patients with early SIRT defined as a SIRT performed within 1 month before or after the first chemotherapy cycle. To finish, landmark analyses of OS at 3 and 6 months were run including patients still alive and followed at 3 and 6 months, respectively, after the initiation of the treatment. The ORR and the secondary surgical resection rates were compared according to SIRT administration with a Wilcoxon test. An univariable logistic regression model was used to estimate the probability of achieving a response (partial or complete) and of undergoing surgical resection. Odds ratios (OR) and 95% CIs were provided.

A propensity score method was used to limit potential bias due to confounding parameters between chemo-SIRT and chemo-only patients for survival, ORR and resection rate outcomes. Univariable logistic regression was first used to model the probability of receiving SIRT and variables with *a p* value <0.1 were introduced into the multivariable model. The AUC and Hosmer–Lemeshow test statistic were estimated to assess model fit. Two approaches using the propensity score were used. Firstly, the inverse probability of treatment weighting (IPTW) method was applied in a Cox regression model to assess the association between treatment and survival, and in a logistic regression model to evaluate treatment response and surgical resection. Secondly, a 1:1 matching population was selected using the caliper method. Covariate balance was calculated using standardized differences before and after propensity score matching, and survival curves were estimated for the matched population.

To identify other prognostic factors for survival, a Cox multivariable regression model was performed. Variables with a *p* value of 0.10 or less in the Cox univariate analysis were considered for inclusion in the multivariable model.

A *p* value of less than 0.05 was considered statistically significant, except for interaction test in subgroup analyses, where a *p* value <0.1 was considered as statistically significant. All statistical tests were two-sided, and *p* values were not adjusted for multiple testing. All analyses were performed using SAS software version 9.3 (SAS Institute, Cary, NC) and R software version 4.1.

## Results

### Patient characteristics

Out of the 2,237 patients included in the ACABi-PRONOBIL cohort, 277 treated between July 2007 and December 2023 met the inclusion criteria of the Real-SIRTCCA study. Of these, 88 patients were included in the chemo-SIRT group and 189 in chemo-only group (Fig. S1). The median follow-up periods were 92.6 (95% CI, 28.6-92.6) and 37.9 months (95% CI, 29.4-65.1), respectively. Baseline patient characteristics are detailed in Table 1. The median age was 65 (IQR 57-73) in the chemo-SIRT group and 64 years (IQR 56-71) in the chemo-only group (*p* = 0.517). The ECOG performance status was 0/1/2 in 60.7%, 34.5%, and 4.8% of patients in the chemo-SIRT group, respectively, and 38.6%, 61.4%, and 0% in the chemo-only group (*p <*0.0001). Cirrhosis was present in 27.3% in the chemo-SIRT group and in 12.8% in the chemo-only group (*p* = 0.003). Regarding tumor characteristics in chemo-SIRT *vs*. chemo-only patients, the median size of the largest tumor was 76.0 *vs.* 65.5 mm (*p* = 0.134), macrovascular invasion and multifocal disease were observed in 24.1 *vs.* 22.6% (*p* = 0.785) and 54.1 *vs.* 58.5% (*p* = 0.497) respectively. Bilobar diseases were observed in 50.6 *vs.* 47.3% (*p* = 0.618), corresponding to centrohepatic single tumors in 9.5% and 9.0% of patients in the chemo-SIRT and chemo-only groups. Liver invasion >50% was present in 9.8% of the chemo-SIRT group compared to 0% in the chemo-only group (*p <*0.0001), as defined by the inclusion criteria. Extrahepatic spread was present in 26.7 *vs.* 34.4% of chemo-SIRT *vs.* chemo-only patients (*p* = 0.208) corresponding to distant lymph-node extension (19.3%) and metastases (11.4%) in the chemo-SIRT group while only lymph-node extension was present in the chemo-only group. Chemotherapy regimens were CISGEM, CISGEM-durvalumab, and GEMOX in 70.5%, 5.7%, and 23.9% of the chemo-SIRT group, respectively, *vs.* 52.4%, 4.8%, and 42.9% of the chemo-only group (*p* = 0.002). The median number of chemotherapy cycles was six in both treatment groups (*p* = 0.777). The delay between the first cycle of chemotherapy and SIRT was 20.1 days (range: 4.6-101.0; Table S1).

**Table 1:** Patient baseline characteristics.

	Overall cohort	Chemo-SIRT	Chemo-only	*p* value
	N = 277	n = 88	n = 189	
**Characteristics**				
Sex, n (%)				
Male	143 (51.6)	41 (46.6)	102 (54.0)	0.253
Female	134 (48.4)	47 (53.4)	87 (46.0)	
Age, median (Q1-Q3)	64 (56.5-71)	65 (57-73)	64 (56-71)	0.517
Missing	1	1	0	
ECOG PS, n (%)				
0	124 (45.4)	51 (60.7)	73 (38.6)	<0.0001
1	145 (53.1)	29 (34.5)	116 (61.4)	
>1	4 (1.5)	4 (4.8)	0 (0.0)	
Missing	4	4	0	
Presence of cirrhosis, n (%)				
Yes	48 (17.5)	24 (27.3)	24 (12.8)	0.003
No	227 (82.6)	64 (72.7)	163 (81.2)	
Missing	2	0	2	
Prior surgery, n (%)	23 (9.3)	5 (6.5)	18 (10.6)	0.305
Missing	30	11	19	

**Extent of disease**				
Tumor size, mm (Q1-Q3)	70 (45.5-98.5)	76.0 (54.5-95.5)	65.5 (40.0-99.5)	0.134
Missing	53	8	45	
Multifocal, n (%)	156 (57.1)	46 (54.1)	110 (58.5)	0.497
Missing	4	3	1	
Bilobar, n (%)	133 (48.4)	44 (50.6)	89 (47.3)	0.618
Missing	2	1	1	
Liver invasion >50%, n (%)	8 (2.9)	8 (9.8)	0 (0.0)	<0.0001
Missing	6	6	0	
Macrovascular invasion, n (%)	62 (23.1)	20 (24.1)	42 (22.6)	0.785
Missing	8	5	3	
Extrahepatic spread, n (%)	88 (32.0)	23 (26.7)	65 (34.4)	0.208
Distant lymph nodes	82 (29.6)	17 (19.3)	65 (34.4)	
Metastases∗	10 (3.6)	10 (11.4)	0 (0.0)	
Missing	2	2	0	

**Tumor grade**, n (%)				0.614
Low	41 (22.2)	15 (25.4)	26 (20.6)	
Intermediate	80 (43.2)	27 (45.8)	53 (42.1)	
High	42 (22.7)	10 (17.0)	32 (25.4)	
Unevaluable	22 (11.9)	7 (11.0)	15 (11.9)	
Missing	92	29	63	

**Type of chemotherapy**, n (%)				0.002
CISGEM/CISGEM-durvalumab	161 (58.1)/14 (5.3)	62 (70.5)/5 (5.7)	99 (52.4)/9 (4.8)	
GEMOX	102 (36.8)	21 (23.9)	81 (42.9)	
Number of cycles, median (Q1-Q3)	6 (4-10)	6 (5-8)	6 (4-11)	0.777

**Biological characteristics**				
CA19.9 (IU/ml), median (Q1-Q3)	92.5 (21.0-686.5)	50.0 (16.0-262.0)	127.0 (24.0-1,925.0)	0.014
Missing	105	19	86	
Bilirubin (μmol/L), median (Q1-Q3)	11.0 (7.5-19.2)	11.4 (7.9-16.1)	10.9 (7.2-21.0)	0.749
Missing	109	22	87	
Albumin (g/L), median (Q1-Q3)	39.0 (34.0-43.0)	40.0 (35.4-43.0)	36.5 (33.0-43.0)	0.125
Missing	125	22	103	

CISGEM, gemcitabine-cisplatin; ECOG PS, Eastern Cooperative Oncology Group performance status; GEMOX, gemcitabine-oxaliplatin; SIRT, selective internal radiotherapy.

Statistical analysis: Levels of significance: *p <*0.05. Wilcoxon test for continuous variables and the chi-square test or Fisher’s exact test for categorical variables.

∗ Other metastatic localizations were bones (n = 3), lung (n = 6) and peritoneal (n = 3).

### SIRT and PFS

**Fig. 1:**
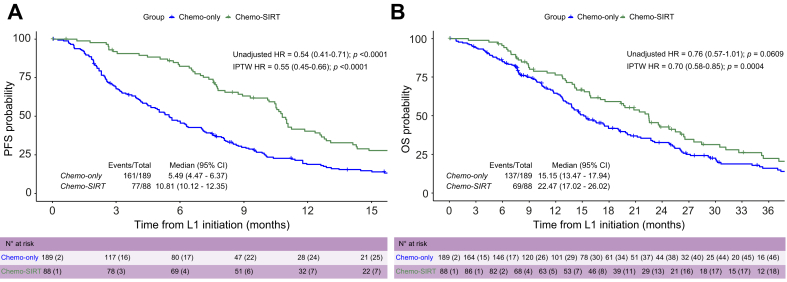
Kaplan-Meier curves for progression-free and overall survival. Statistical analysis: Levels of significance: *p* <0.05. Kaplan-Meier method to estimate the association between treatment group and (A) PFS and (B) OS, described with medians and 95% CI. Univariable Cox regression to assess HRs with 95% CIs for unadjusted estimation and corrected estimation with the IPTW method. *p* values are provided from Cox models. Chemo, chemotherapy, HR, hazard ratio; IPTW, inverse probability of treatment weighting; OS, overall survival; PFS, progression-free survival; SIRT, selective internal radiation therapy.

Unadjusted median (m)PFS was significantly longer in the chemo-SIRT group: 10.8 months (95 CI% 10.1-12.4) compared to 5.5 months (95 CI% 4.5-6.4) in the chemo-only group (HR 0.54, 95% CI 0.41-0.71, log-rank *p <*0.0001; Fig. 1A). As the proportionality assumption was violated, a restricted mean analysis was performed. The adjusted difference in restricted mean survival time was 5.1 months (95% CI 2.6-7.6; *p <*0.001).

**Fig. 2:**
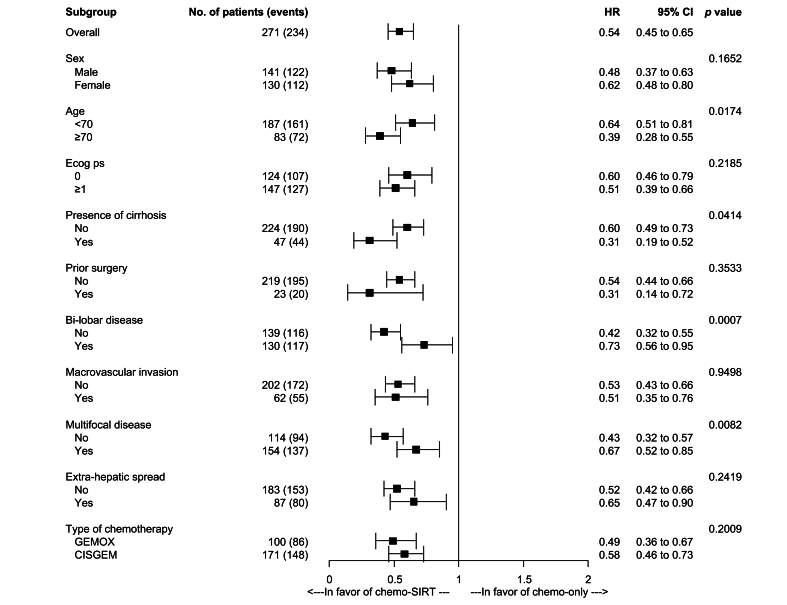
Forest plots of progression-free survival in clinically relevant subgroups for chemotherapy-SIRT *vs.* chemotherapy only in the IPTW univariable Cox regression model. Statistical analysis: Levels of significance: *p* <0.1. IPTW method applied in univariable Cox regression to assess the association between treatment group and PFS in each subgroup. The cox models include the treatment, the group and the interaction term between group and treatment. The *p* values provided correspond to the interaction term *p* value. CISGEM, cisplatin-gemcitabine; ECOG PS, Eastern Cooperative Oncology Group performance status; GEMOX, gemcitabine-oxaliplatin; HR, hazard ratio; IPTW, inverse probability of treatment weighting; SIRT, selective internal radiation therapy.

The propensity score was constructed with all relevant variables that were unbalanced between patients receiving chemo-SIRT and patients receiving chemo-only. Multivariable logistic regression, including the presence of cirrhosis, ECOG performance status, and type of chemotherapy, estimated the probability of being in the chemo-SIRT group, with an AUC of 0.67 (Table S2). A 1:1 matched population using a caliper of 0.15 was built, resulting in 77 matched pairs. After propensity score matching, most differences between the groups were reduced (Fig. S2). Following 1:1 matching, chemo-SIRT was associated with a significant increase in PFS, with a median of 10.6 months compared to 5.6 months in the chemo-only group (HR 0.54, 95% CI 0.38-0.77, *p* = 0.0006; Fig. S3A). Finally, IPTW analysis confirmed the superiority of chemo-SIRT in PFS (HR 0.55, 95% CI 0.45-0.66, *p <*0.0001; Fig. 1A), with a benefit generally consistent across clinically meaningful subgroups analyzed (Fig. 2). The sensitivity analysis in patients receiving early SIRT (n = 50) confirmed the superiority of the SIRT strategy with an unadjusted mPFS of 12.6 *vs*. 5.5 months (unadjusted HR 0.48, 95% CI 0.34-0.68, *p <*0.0001; HR after IPTW 0.44, 95% CI 0.36-0.53, *p <*0.0001) (Fig. S4A).

### SIRT and secondary objectives

Unadjusted mOS was 22.5 months in the chemo-SIRT group compared to 15.1 months in the chemo-only group (HR 0.76, 95% CI 0.57-1.01, *p* = 0.061; Fig. 1B). After 1:1 matching, mOS with chemo-SIRT was 22.5 months compared to 14.8 months in the chemo-only group (HR 0.74, 95% CI 0.51-1.07, *p* = 0.110; Fig. S3B), with significant superiority of the combination after IPTW (HR 0.70, 95% CI 0.58-0.85, *p* = 0.0004; Fig. 1B). The OS benefit of chemo-SIRT was generally consistent across clinically meaningful subgroups analyzed (Fig. S6). The sensitivity analysis in patients receiving early SIRT confirmed the results of the overall cohort with an unadjusted mOS of 22.5 *vs.* 15.1 in the chemo-SIRT and chemo-only groups, respectively (HR 0.84, 95% CI 0.59-1.19, *p* = 0.317), with significant superiority of the combination after IPTW (HR 0.75, 95% CI 0.61-0.92, *p* = 0.007) (Fig. S4B). Landmark analysis of OS at 3 months confirmed the superiority of the association after IPTW with a mOS of 19.5 *vs*. 13.3 months in chemo-SIRT *vs.* chemo-only groups (unadjusted HR 0.79, 95% CI 0.559-1.07, *p* = 0.123), reaching statistical significance after IPTW (HR 0.75, 95% CI 0.61-0.92, *p* = 0.005*)* (Fig. S5A)*.* Similarly, at 6 months, mOS was 16.5 *vs*. 11.9 in chemo-SIRT *vs.* chemo-only groups (unadjusted HR 85, 95% CI 0.63-1.16, *p* = 0.301; IPTW-HR 0.81, 95% CI 0.65-1.00, *p* = 0.05) (Fig. S5B).

Unadjusted ORR was higher in the chemo-SIRT group at 58.3% compared to 28.5% in the chemo-only group (OR 3.51, 95% CI 2.03-6.09, *p <*0.0001). This superiority of chemo-SIRT was confirmed after IPTW analysis (OR 3.17, 95% CI 2.18-4.49, *p <*0.0001; Table 2).

**Table 2:** Objective response rate according to RECIST 1.1.

	Chemo-SIRT	Chemo-only	Overall cohort	*p* values
	n = 88	n = 189	N = 277	
Best overall response, n (%)				
Complete	6 (7.1)	0 (0.0)	6 (2.4)	<0.0001
Partial	43 (51.2)	47 (28.5)	90 (36.1)	
Stable	27 (32.1)	63 (38.2)	90 (36.1)	
Progression	8 (9.5)	55 (33.3)	63 (25.3)	
Missing	4	24	28	
Objective response rate∗, n (%)	49 (58.3)	47 (28.5)	96 (38.6)	<0.0001
Disease control rate∗∗, n (%)	76 (90.5)	110 (66.7)	186 (74.7)	0.0001

SIRT, selective internal radiotherapy.

Statistical analysis: Levels of significance: *p* <0.05. Chi-square test.

∗ Includes complete and partial response.

∗∗ Includes complete, partial, and stable response.

Secondary surgical resection of the primary tumor was significantly more frequent in the chemo-SIRT group, being performed in 18.7% of patients including two liver transplants *vs.* 8.8% in the chemo-only group (unadjusted OR 2.37, 95% CI 1.08-5.21, *p* = 0.0314; IPTW OR 2.94, 95% CI 1.71-5.03, *p <*0.0001).

All-grade and grade 3-4 adverse events were observed in 50.8% and 21.0% of patients in the chemo-SIRT group, respectively, and 76.1% and 20.4% of patients in the chemo-only group (Table 3).

**Table 3:** Adverse events according to treatment.

	Chemo-SIRT	Chemo-only	Overall population	*p* value
	n = 88	n = 189	N = 277	
Adverse event, n (%)				
Any grade	32 (50.8)	118 (76.1)	150 (68.8)	0.107
Grade 3-4	13 (21.0)	31 (20.4)	44 (20.6)	0.925
Missing	25	34	59	
Hematological toxicity, n (%)				
Any grade	22 (34.9)	87 (56.1)	109 (50.0)	0.005
Grade III-IV	12 (19.1)	24 (15.6)	36 (16.6)	0.534
Missing	25	34	59	
Neuropathy, n (%)				
Any grade	10 (16.1)	57 (37.0)	67 (31.0)	0.003
Grade III-IV	1 (1.7)	6 (3.9)	7 (3.3)	0.676
Missing	26	35	61	
Digestive toxicity, n (%)				
Any grade	10 (16.4)	82 (53.6)	92 (43.0)	<0.0001
Grade III-IV	2 (3.3)	7 (4.6)	9 (4.2)	1.00
Missing	27	36	63	
Infectious complications, n (%)				
Any grade	3 (4.9)	9 (5.9)	12 (5.6)	1.00
Missing	27	37	64	

SIRT, selective internal radiotherapy.

Statistical analysis: Levels of significance: *p* <0.05. Chi-square test or Fisher’s exact test.

### Prognostic factors in the overall population

In multivariable Cox regression analyses, chemo-SIRT was the only factor associated with significantly longer PFS (HR 0.55, 95% CI 0.41-0.74, *p <*0.0001) (Table S3).

For OS, chemo-SIRT was associated with significantly longer OS (HR 0.72, 95% CI 0.53-0.98, *p* = 0.004). and other independent factors associated with longer OS were ECOG PS 0 and the absence of cirrhosis (Table S4).

## Discussion

Real-SIRTCCA is the largest cohort study comparing first-line chemotherapy for advanced iCCA with or without SIRT in the real-life setting. The addition of concurrent SIRT to first-line chemotherapy improved efficacy outcomes in patients with locally advanced or limited extrahepatic tumor burden. Univariate analysis showed a significant increase of mPFS for the chemo-SIRT group, which was confirmed by IPTW propensity score analysis (HR 0.55, *p <*0.0001). Although OS was not statistically significantly longer in the chemo-SIRT group before adjustment, the combination significantly improved this outcome after IPTW adjustment (HR 0.70, *p* = 0.0004). This benefit in OS was confirmed by landmark analyses at 3 and 6 months after IPTW (3-month HR 0.75, *p* = 0.005; 6-month HR 0.81, *p* = 0.05). Additionally, local tumor control and ORR were significantly better with chemo-SIRT (*p <*0.0001). These results reinforce the assumption that intrahepatic locoregional treatment through multi-modal strategies improve patient survival outcomes, likely due to better control of the liver tumor burden, which can impair prognosis.^10^ Furthermore, the improved tumor response suggests that chemo-SIRT may help downstage patients and make them eligible for more aggressive treatments, such as intent-to-cure surgery, as evidenced by the significant higher rate of secondary tumor resection or liver transplantation in the chemo-SIRT group (18.7% *vs.* 8.8%) before and after adjustment. Besides, the safety profile of the combination was comparable, with both groups receiving a median of six chemotherapy cycles and no additional toxicity related to SIRT.

To date, there are only retrospective or non-randomized data suggesting the potential benefit of hepatic intra-arterial strategies for iCCA. A large meta-analysis, which included 1,232 patients treated with SIRT from 27 non-controlled, retrospective cohorts, reported mPFS and mOS of 7.8 and 14.1 months, respectively. These outcomes did not differ from survival rates in patients with locally advanced iCCA who did not have access to SIRT.^19^ However, this meta-analysis noted considerable heterogeneity in technique, systemic therapies, and the lack of systematic personalized dosimetry. Although only 63 patients from four different cohorts received concurrent chemotherapy with SIRT, pooled analyses of all intra-arterial treatments suggested a greater benefit in OS, PFS, and response rate when these procedures were performed combined with first-line systemic therapy.^19^ The only randomized phase III trial to date that evaluated SIRT in iCCA is SIRCCA (NCT02807181), which compared SIRT followed by CISGEM to CISGEM alone. Nonetheless, this trial was stopped prematurely due to insufficient recruitment rate, and no results have been reported yet. The single-arm phase II MISPHEC trial provided significant insights into the efficacy of the chemo-SIRT combination for iCCA. This study showed potential benefits for patients with locally advanced iCCA, with a 3-month ORR (primary outcome) of 39% (95% CI 26%-53%), mPFS of 14 months (95% CI 8-17 months), and mOS of 22 months (95% CI 14-52 months). It included 41 patients with iCCA and limited or no extrahepatic disease (hilar lymph node ≤3 cm or <5 lung nodules, each ≤10 mm).^15^ These promising results were further reinforced by a pooled analysis of patients from MISPHEC compared to controls treated with chemotherapy alone from five prospective trials (ABC-01,^20^ ABC-02,^7^ ABC-03,^21^ BINGO,^22^ and PRODIGE38-AMEBICA^23^). This meta-analysis showed that combining first-line chemotherapy with SIRT significantly improved PFS (8.4 *vs.* 4.3 months; HR 0.52 95% CI 0.31-0.89, *p <*0.001) and OS (21.7 vs 15.9 months; HR 0.59 95% CI 0.34-0.99, *p* = 0.049) compared to chemo-alone.^24^ This pooled analysis was the first study to demonstrate the benefit of the combination over systemic therapy alone. However, due to the scarcity of prospective data specific to locally advanced iCCA, the results were based on a limited number of patients (n = 84), with selection biases in the control arm potentially arising from the ineligibility of patients for SIRT. Furthermore, none of these trials offered a direct, face-to-face comparison of the two strategies.^24^ Our study confirmed these findings in a larger cohort of patients treated in a real-life setting across France. Nevertheless, due to its retrospective nature, some limitations remain, such as selection biases between the two treatment groups. These biases stem from the challenge of ensuring that control patients would have been eligible for SIRT, which requires a systemic pre-SIRT work-up phase, combining arteriography and dosimetry studies. To limit these biases, we implemented a robust selection strategy for the control group, systematically excluding patients with an ECOG performance status >1, visceral extrahepatic spread, or liver involvement >50% as these do not align with typical SIRT candidates. Patients with missing data related to these criteria were also excluded. In the chemo-SIRT group, the eligibility criteria were less strict, in accordance with MISPHEC’s criteria,^15^ to allow a real-life assessment of SIRT outcomes based on routine care practice from participating centers. This differential selection may have let to the inclusion of more advanced cases, such as those with liver tumor involvement >50% or limited extrahepatic visceral metastases, as well as more underlying cirrhosis in the chemo-SIRT group (27.3% *vs.* 12.8%). The overrepresentation of cirrhosis in the chemo-SIRT group might also be explained by the fact that hepatocellular insufficiency and/or portal hypertension made locoregional therapies combined with chemotherapy preferable to upfront major surgery, leading to a higher proportion of potentially resectable disease with cirrhosis. The groups were comparable in terms of prognostic markers such as lymph-node extension, multifocal, bilobar disease, and macrovascular invasion, all of which could influence intrahepatic treatments. Moreover, the same proportion of centro-hepatic tumors was observed in the two groups even though this type of disease might favor the chemo-SIRT strategy. Besides, ECOG performance status of 0 was significantly more frequent in the chemo-SIRT group, which might potentially affect the comparability of groups before adjustment. Since SIRT is routinely performed in only a few expert centers in France and is not yet a standard treatment, it was unlikely that control patients were those initially considered for SIRT but later deemed ineligible. Additionally, most control patients were from centers that did not perform SIRT or were treated before SIRT reimbursement began in 2018.

As Real-SIRTCCA is a real-life observational study, there was potential heterogeneity in treatment schedules. First, regarding systemic therapy, CISGEM was used more in the chemo-SIRT group (76.1 *vs.* 57.1% of patients). GEMOX was an alternative in the first-line setting^12^^,^^25^^,^^26^ before the validation of FOLFOX (folinic acid, fluorouracil and oxaliplatin) in the second-line setting in 2020.^27^ Although no differences in the inclusion period were observed between groups (Fig. S7), CISGEM might have been more often preferred in the chemo-SIRT group due to the influence of the MISPHEC French trial, which started in 2013 and was published in 2020.^15^ The low number of patients receiving CISGEM-durvalumab, due to its recent availability in France (November 2022), did not allow for statistical comparison. Moreover, the SIRT procedure was not standardized, and there was no systematic personalized dosimetry, which might have decreased the efficacy of SIRT due to an insufficient dose delivered to the tumor, potentially affecting its safety. Indeed, the median dose delivered to the tumor in Real-SIRTCCA was lower to that in MISPHEC (Table S1). The timing between the initiation of chemotherapy and SIRT was also not standardized, but with a median of 20.1 days, it was close to the MISPHEC schedule, where SIRT was performed during the first CISGEM cycle. Additionally, 75% of patients in Real-SIRTCCA had SIRT within the first 3 months of chemotherapy, which is pragmatic, as SIRT organization usually takes place during the first months of chemotherapy. This also presents a limitation, as some patients might have had their first computed tomography scan assessment under chemotherapy before SIRT, potentially modifying their treatment strategy and compromising the similarity between groups due to retrospective selection. However, sensitivity analyses in the 50 patients receiving early SIRT defined as a SIRT within 1 month before or after the first chemotherapy cycle confirmed the benefit of the strategy with better survival outcomes. Another limitation due to the retrospective collection of some data, is the proportion of missing baseline biological characteristics, as well as the comparison of treatment safety, since adverse events are usually less well-documented in this type of cohort. Yet, no differences were observed among patients for whom these data were available. Finally, although it should not interfere with group comparability, cohorts such as PRONOBIL often suffer from reporting bias, with an overrepresentation of expert centers, making them less representative of the nationwide therapeutic landscape. Indeed, these findings need to be viewed in the context of the fact that SIRT requires sophisticated technique platforms and remains inaccessible to many patients, even in Western countries.

To date, the use of SIRT in iCCA remains controversial as guidelines such as EASL-ILCA^14^ consider it as an alternative to systemic therapy in unresectable diseases, while ESMO recommends it as a first-line treatment standard.^11^ However, AASLD considers it as a technique with insufficient data to be broadly recommended.^13^ Prospective randomized data are urgently needed to advance the field, but it is unlikely that such data will become available. Indeed, to properly assess SIRT benefit, a phase II or III randomized trial should be run comparing CISGEM plus immunotherapy with or without SIRT in non-resectable iCCA. Liver-only and oligometastatic diseases should be included and stratified with less strict inclusion criteria than in SIRCCA to ensure a sufficient recruitment and correspond to real-life need. However, such a trial should be international and would be complex to run due to the risk of drop-out after the SIRT work-up phase and the scarcity of the disease.

The strengths of this cohort include its multicentric, ambispective character, supported by the PRODIGE intergroup, offering a nationwide overview of practices in iCCA treatment. This is the largest cohort to date of patients treated with the chemo-SIRT combination compared with control patients selected through a robust process. Given that Real-SIRTCCA demonstrated a great improvement in survival outcomes, tumor response and secondary tumor resection rates in favor of chemo-SIRT, we believe that SIRT should be considered for selected patients with locally advanced, unresectable iCCA who are eligible for first-line chemotherapy with CISGEM-immunotherapy and have limited or no extrahepatic tumor burden. The safety and potential efficacy of SIRT, combined with systemic chemotherapy, whether as a bridge to intent-to-cure strategies or in a palliative setting, should encourage the adoption of this therapeutic strategy in a larger panel of clinical centers, or at very least, prompt clinicians to refer their patients to centers where SIRT is performed.

Real-SIRTCCA suggests that concurrent SIRT with standard first-line chemotherapy improves survival outcomes, with longer PFS and OS, as well as provides better ORRs and rates of secondary tumor resection, without additional toxicity in patients with unresectable iCCA who have limited or no extrahepatic tumor burden. Although these findings ideally require confirmation through prospective randomized trials, this study constitutes new evidence supporting the potential benefit of this strategy in selected patients.

## Abbreviations

CISGEM, cisplatin-gemcitabine; ECOG, Eastern Cooperative Oncology Group; GEMOX, gemcitabine-oxaliplatin; HR, hazard ratio; iCCA, intrahepatic cholangiocarcinoma; IPTW, inverse probability of treatment weighting; OR, odds ratio; ORR, objective response rate; OS, overall survival; PFS, progression-free survival; SIRT, selective internal radiation therapy.

## Financial support

The GERCOR G-115 PRODIGE 83 ACABi-PRONOBIL study was supported by GERCOR and the ARCAD foundation (Aide et Recherche en Cancerologie Digestive) and funded by ACABi (Association pour l'étude des Cancers et Affections des voies Biliaires).

## Authors’ contributions

Study concept and design: NA, JH, DV, GR. Acquisition of data: NA, JE, NF, TL, AT, MV, BC, DT, CT, EA, MD, SM, OB, NW, AV, LB, LM, CN, AB, GR. Analysis and interpretation of data: NA, JH, DV, GR. Drafting of the manuscript: NA, JH, DV, GR. Critical revision of the manuscript for important intellectual content: NA, JE, NF, TL, AT, MV, BC, DT, CT, EA, MD, SM, OB, NW, AV, LB, LM, CN, AB, GR. Statistical analysis: JH, DV. Obtained funding: AT, CN. Administrative technical, or material support: AT, CN. Study supervision: GR.

## Data availability statement

All data associated with this study are present within the main text or in the Supplementary material.

## Ethics approval

Non-opposing forms were required for living patients as mandated by the ACABi-PRONOBIL ethics committee. The study was approved by the Comité de Protection des Personnes Est III on April 23, 2024, and complies with the French MR003 methodology regarding general data protection regulation for non-interventional retrospective health research (N°ID-RCB:2021-A00835-36).

## Conflict of interest

Nicolas Adamus declares no conflict of interest. Julien Edeline declares Consulting: MSD, Eisai, BMS, AstraZeneca, Bayer, Roche, Ipsen, Basilea, Merck Serono, Incyte, Servier, Beigene, Taiho, Boston Scientific, Guerbet; Travel expense from Amgen; Research funding (institutional) from BMS, Beigene, Boston Scientific, Exeliom biosciences, SUMMIT. Julie Henriques declares no conflict of interest. Nadim Fares declares consulting and/or advisory role and/or invitation as a speaker for Servier, AstraZeneca, Incyte, as well as travel accommodations, or expenses from Servier, AstraZeneca. Research funding by Servier. Thierry Lecomte declares honoraria for speaking or consulting role from Astra Zeneca, Incyte. Anthony Turpin has received personal fees from Servier, Viatris, Incyte Bioscience, BMS, Merck and grants and personal fees from AstraZeneca and MSD outside the submitted work. Dewi Vernerey declares consulting fees for OSE Imminotherapeutics, Janssen-Cilag, HalioDx, cellprothera, GERCOR, Incyte, FSK, Invectys, AC biotech, Veracyte, CURE51, Apmonia Therapeutics. Mathilde Vincens declares no conflict of interest. Brice Chanez no conflict of interest related to this work. Christophe Tournigand declares no conflict of interest. Eric Assenat declares consulting and/or advisory role and/or invitation as a speaker for Servier, AstraZeneca, Incyte, Roche, BMS as well as travel accommodations, or expenses from Servier, Roche, and AstraZeneca. Research funding by Roche. Matthieu Delaye declares no conflict of interest. Cindy Neuzillet declares consulting and/or advisory role and/or invitation as a speaker for: Amgen, Astellas, AstraZeneca, AAA, Baxter, Bristol-Myers Squibb, Boehringer Ingelheim, Fresenius Kabi, Incyte Biosciences, Merck, MSD, Mundipharma, Nestlé Health Science, Novartis, Nutricia, OSE Immunotherapeutics, Pierre Fabre, Roche, Sanofi, Servier, Viatris; research funding (to institution): AstraZeneca, Bristol-Myers Squibb, Fresenius Kabi, Nutricia, OSE Immunotherapeutics, Roche, Servier, Viatris. Sylvain Manfredi declares no conflict of interest. Olivier Bouché declares consulting and/or advisory role and/or invitation as a speaker for Amgen, Astellas, Bayer, Merck Serono, Servier, Pierre Fabre, Takeda, Deciphera, and MSD, as well as travel accommodations, or expenses from MSD, Pierre Fabre, and Servier. Nicolas Williet declares honoraria/consulting fees for Accord Healthcare, Astrazeneca, Ipsen, Lac Leo Pharma, Mayoly, MSD, Pierre Fabre, Servier, Viatris. Research funding/clinical trials: None. Angelique Vienot declares no conflict of interest. Lorraine Blaise declares no conflict of interest. Léo Mas declares consulting and/or advisory role for Merck Serono. David Tougeron declares consulting and/or advisory role and/or invitation as a speaker for Servier, AstraZeneca, Bristol-Myers Squibb, MSD, Amgen, Pierre Fabre, Incyte, Merck Serono, Takeda and Roche, as well as travel accommodations, or expenses from Servier, MSD, Roche, Amgen, and Pierre Fabre. Research funding by MSD, Roche, AstraZeneca, Servier and Pierre Fabre. Alice Boilève declares no conflict of interest. Gael Roth declares consulting and/or advisory role and/or invitation as a speaker for Servier, AstraZeneca, Bristol-Myers Squibb, MSD, Amgen, Ipsen, Pierre Fabre, Incyte, Netris Pharma and Alpha Tau, as well as travel accommodations, or expenses from Servier, AstraZeneca, Bristol Myers Squibb, MSD, Roche, Amgen, Viatris, Pierre Fabre and Ipsen. Research funding by Genoscience Pharma, Netris Pharma.

Please refer to the accompanying ICMJE disclosure forms for further details.
