# Supplementary material for: First-line chemotherapy with selective internal radiation therapy for intrahepatic cholangiocarcinoma: The French ACABi GERCOR PRONOBIL cohort
Source: JHEP Rep. 2024 Nov 20;7(2):101279. doi: 10.1016/j.jhepr.2024.101279 (PMC11786833; doi:10.1016/j.jhepr.2024.101279)
Supplement: Multimedia component 1 [file mmc1.pdf]

# First-line chemotherapy with selective internal radiation therapy for intrahepatic cholangiocarcinoma: The French ACABi GERCOR PRONOBIL cohort

Nicolas Adamus, Julien Edeline, Julie Henriques, Nadim Fares, Thierry Lecomte, Anthony Turpin, Dewi Vernerey, Mathilde Vincens, Brice Chanez, David Tougeron, Christophe Tournigand, Eric Assenat, Matthieu Delaye, Sylvain Manfredi, Olivier Bouché, Nicolas Williet, Angelique Vienot, Lorraine Blaise, Léo Mas, Cindy Neuzillet, Alice Boilève, Gaël S Roth

Table of contents

Table S1……………………………………………………………………………………2

Table S2……………………………………………………………………………………3

Table S3……………………………………………………………………………………5

Table S4……………………………………………………………………………………6

Fig. S1……………………………………………………………………………………..8

Fig. S2……………………………………………………………………………………..9

Fig. S3……………………………………………………………………………………10

Fig. S4……………………………………………………………………………………11

Fig. S5……………………………………………………………………………………12

Fig. S6……………………………………………………………………………………13

Fig. S7……………………………………………………………………………………14

**Table S1. SIRT characteristics.**

| | Chemo-SIRT, n = 88 |
|---|---|
| **Nuclear activity injected** (Gbq) | |
| Median (Q1-Q3) | 2.0 (1.4-3.1) |
| Missing | 15 |
| **Dose delivered to the tumor** (Gy) | |
| Median (Q1-Q3) | 192.5 (122.5-259.0) |
| Missing | 32 |
| **Dose delivered to the targeted liver** (Gy) | |
| Median (Q1-Q3) | 146.0 (114.0-270.0) |
| Missing | 43 |
| **Dose delivered to the total liver** (Gy) | |
| Median (Q1-Q3) | 69.0 (46.5-86.5) |
| Missing | 44 |
| **Pulmonary shunt** (%*) | |
| Median (Q1-Q3) | 0.7 (0.0-1.0) |
| Missing | 48 |
| **Delay between chemotherapy initiation and SIRT** (days) | |
| Median (Q1-Q3) | 18 (3-99) |
| Missing | 0 |

**Abbreviations:** SIRT: selective internal radiotherapy.
*Percentage of the total injected dose found in the lungs during work-up phase.

**Table S2. Univariate and multivariate Cox regression analyses of progression-free survival.**

| | Univariate analysis | | | Multivariate analysis | | |
|---|---|---|---|---|---|---|
| | N (events) | HR (CI 95%) | *p*-value | N (events) | HR (CI 95%) | *p*-value |
| **Sex** | | | | | | |
| Male | 143 (122) | 1.00 | | | | |
| Female | 134 (116) | 0.95 (0.74-1.23) | 0.702 | | | |
| **Age** | | | | | | |
| <70 | 191 (164) | 1.00 | | | | |
| >70 | 85 (73) | 1.02 (0.78-1.35) | 0.868 | | | |
| **ECOG PS** | | | | | | |
| 0 | 124 (107) | 1.00 | | 123 (89) | 1.00 | |
| >=1 | 149 (128) | 1.30 (1.00-1.68) | 0.050 | 147 (113) | 1.13 (0.87-1.48) | 0.369 |
| **Presence of cirrhosis** | | | | | | |
| Yes | 48 (45) | 1.00 | | | | |
| No | 227 (192) | 0.77 (0.55-1.07) | 0.121 | | | |
| **Prior surgery** | | | | | | |
| No | 224 (198) | 1.00 | | | | |
| Yes | 23 (20) | 0.76 (0.47-1.22) | 0.258 | | | |
| **Multifocal disease** | | | | | | |
| No | 117 (96) | 1.00 | | | | |
| Yes | 156 (138) | 0.99 (0.76-1.28) | 0.921 | | | |
| **Bi-lobar disease** | | | | | | |
| No | 142 (118) | 1.00 | | | | |
| Yes | 133 (119) | 1.07 (0.83-1.38) | 0.622 | | | |
| **Liver invasion > 50%** | | | | | | |
| No | 263 (224) | 1.00 | | | | |
| Yes | 8 (8) | 1.07 (0.53-2.17) | 0.854 | | | |
| **Macrovascular invasion** | | | | | | |
| No | 207 (175) | 1.00 | | | | |
| Yes | 62 (55) | 1.00 (0.74-1.36) | 0.983 | | | |
| **Tumor grade** | | | | | | |
| Low | 41 (35) | 1.00 | | | | |
| Intermediate | 80 (72) | 1.21 (0.81-1.82) | 0.325 | | | |
| High | 42 (37) | 1.39 (0.87-2.23) | | | | |
| Not assessable | 22 (15) | 0.86 (0.47-1.58) | | | | |
| **Extrahepatic spread** | | | | | | |
| No | 187 (156) | 1.00 | | 115 (80) | 1.00 | |
| Yes | 88 (80) | 1.38 (1.05-1.81) | 0.02145 | 155 (122) | 0.94 (0.72-1.22) | 0.623 |
| **Type of chemotherapy** | | | | | | |
| GEMOX | 102 (87) | 1.00 | | | | |
| GEMCIS | 175 (151) | 0.82 (0.63-1.07) | 0.146 | | | |
| **Group** | | | | | | |
| CT | 189 (161) | 1.00 | | 188 (136) | 1.00 | |

| | | | | | | |
|---|---|---|---|---|---|---|
| CT+SIRT | 88 (77) | 0.54 (0.41-0.71) | 0.00001 | 82 (66) | 0.55 (0.41-0.74) | <0.0001 |

**Abbreviations**: CISGEM: gemcitabine - cisplatin; CT: chemotherapy; ECOG PS: eastern cooperative oncology group performance status; GEMOX: gemcitabine - oxaliplatin; PFS: progression-free survival; SIRT: selective internal radiation therapy.
**Statistical analysis:** Univariable and multivariable Cox regression to assess the association between PFS and clinical parameters. Variables with P-value <0.1 in univariable models were then included in the multivariable model.The Levels of significance for multivariable model is P-value<0.05.

**Table S3. Univariate and multivariate logistic regression analysis to estimate the probability of belonging to the SIRT group.**

| | N (SIRT) | Univariate OR [CI 95%] | *p*-value | N (SIRT) | Multivariate OR [CI 95%] | *p*-value |
|---|---|---|---|---|---|---|
| **Sex** | | | | | | |
| Male | 143 (41) | 1.00 | | | | |
| Female | 134 (47) | 1.34 (0.81-2.23) | 0.253 | | | |
| **Age (years)** | | | | | | |
| Continuous | 376 (87) | 1.002 (0.98-1.03) | 0.854 | | | |
| **ECOG PS** | | | | | | |
| 0 | 124 (51) | 1.00 | | 124 (51) | 1.00 | |
| >1 | 149 (33) | 0.41 (0.24-0.69) | 0.0008 | 147 (33) | 0.44 (0.26-0.76) | 0.003 |
| **Presence of cirrhosis** | | | | | | |
| No | 227 (64) | 1.00 | | 224 (61) | 1.00 | |
| Yes | 48 (24) | 2.55 (1.35-4.81) | 0.004 | 47 (23) | 2.61 (1.33-5.11) | 0.005 |
| **Prior surgery** | | | | | | |
| No | 224 (72) | 1.00 | | | | |
| Yes | 23 (5) | 0.59 (0.21-1.64) | 0.310 | | | |
| **Multifocal disease** | | | | | | |
| No | 117 (39) | 1.00 | | | | |
| Yes | 156 (46) | 0.84 (0.50-1.41) | 0.497 | | | |
| **Bi-lobar disease** | | | | | | |
| No | 142 (43) | 1.00 | | | | |
| Yes | 133 (44) | 1.14 (0.69-1.89) | 0.618 | | | |
| **Macrovascular invasion** | | | | | | |
| No | 207 (63) | 1.00 | | | | |
| Yes | 62 (20) | 1.09 (0.59-2.00) | 0.785 | | | |
| **Tumor grade** | | | | | | |
| Low | 41 (15) | 1.00 | 0.619 | | | |
| Intermediate | 80 (27) | 0.88 (0.40-1.94) | 0.757 | | | |
| High | 42 (10) | 0.54 (0.21-1.41) | 0.208 | | | |
| Unevaluable | 22 (7) | 0.81 (0.27-2.43) | 0.705 | | | |
| **Extrahepatic spread** | | | | | | |
| No | 187 (63) | 1.00 | 0.209 | | | |
| Yes | 88 (23) | 0.70 (0.40-1.22) | | | | |
| **Type of chemotherapy** | | | | | | |
| GEMOX | 102 (21) | 1.00 | | 100 (20) | 1.00 | |
| GEMCIS | 175 (67) | 2.39 (1.36-4.23) | 0.003 | 171 (64) | 2.40 (1.31-4.38) | 0.004 |

**Abbreviations**: CISGEM: gemcitabine - cisplatin combination; ECOG PS: eastern cooperative oncology group performance status; GEMOX: gemcitabine - oxaliplatin combination; OS: overall survival; SIRT: selective internal radiotherapy.
**Statistical analysis:** Univariable and multivariable logistic regression to assess the probability of belonging to the SIRT group. Variables with P-value <0.15 in univariable models were then included in the multivariable model. The Levels of significance for multivariable model is P-value<0.05.

**Table S4: Univariate and multivariate Cox regression analyses of overall survival.**

| | Univariate analysis | | | Multivariate analysis | | |
|---|---|---|---|---|---|---|
| | N (events) | HR [CI 95%] | p-value | N (events) | HR [CI 95%] | p-value |
| **Sex** | | | | | | |
| Male | 143 (115) | 1.00 | | 141 (115) | 1.00 | |
| Female | 134 (91) | 0.76 (0.58-1) | 0.053 | 130 (89) | 0.82 (0.61-1.09) | 0.172 |
| **Age** | | | | | | |
| <70 | 191 (143) | 1.00 | | | | |
| >70 | 85 (62) | 1.20 (0.89-1.61) | 0.240 | | | |
| **ECOG PS** | | | | | | |
| 0 | 124 (90) | 1.00 | | 124 (90) | 1.00 | |
| >=1 | 149 (115) | 1.47 (1.11-1.94) | 0.007 | 147 (114) | 1.40 (1.05-1.86) | 0.020 |
| **Presence of cirrhosis** | | | | | | |
| Yes | 48 (42) | 1.00 | | 47 (42) | 1.00 | |
| No | 227 (163) | 1.49 (1.06-2.09) | 0.022 | 224 (162) | 1.62 (1.12-2.34) | 0.011 |
| **Prior surgery** | | | | | | |
| No | 224 (174) | 1.00 | | | | |
| Yes | 23 (16) | 0.78 (0.47-1.31) | 0.345 | | | |
| **Multifocal disease** | | | | | | |
| No | 117 (80) | 1.00 | | | | |
| Yes | 156 (122) | 0.94 (0.71-1.25) | 0.664 | | | |
| **Bi-lobar disease** | | | | | | |
| No | 142 (96) | 1.00 | | | | |
| Yes | 133 (108) | 1.13 (0.86-1.49) | 0.391 | | | |
| **Liver invasion > 50%** | | | | | | |
| No | 263 (192) | 1.00 | | | | |
| Yes | 8(8) | 1.07 (0.53-2.17) | 0.855 | | | |
| **Macrovascular invasion** | | | | | | |
| No | 207 (152) | 1.00 | | | | |
| Yes | 62 (46) | 0.95 (0.68-1.33) | 0.782 | | | |
| **Tumor grade** | | | | | | |
| Low | 41 (31) | 1.00 | | | | |
| Intermediate | 80 (55) | 0.93 (0.59-1.44) | 0.893 | | | |
| High | 42 (34) | 1.08 (0.66-1.75) | | | | |
| Unevaluable | 22 (14) | 1.10 (0.58-2.07) | | | | |
| **Extra-hepatic spread** | | | | | | |
| No | 187 (63) | 1.00 | | | | |
| Yes | 88 (23) | 0.70 (0.40-1.22) | 0.2087 | | | |
| **Chemotherapy characteristics** | | | | | | |
| GEMOX | 102 (80) | 1.00 | | | | |
| GEMCIS | 175 (126) | 0.85 (0.64-1.12) | 0.247 | | | |
| **Group** | | | | | | |
| CT | 189 (137) | 1.00 | | 187 (136) | 1.00 | |

| | | | | | | |
|---|---|---|---|---|---|---|
| CT+SIRT | 88 (69) | 0.76 (0.57-1.01) | 0.061 | 84 (68) | 0.73 (0.53-0.99) | 0.004 |

**Abbreviations**: CISGEM: gemcitabine - cisplatin; CT: chemotherapy; ECOG PS: eastern cooperative oncology group performance status; GEMOX: gemcitabine - oxaliplatin; OS: overall survival; SIRT: selective internal radiation therapy.
**Statistical analysis:** Univariable and multivariable Cox regression to assess the association between OS and clinical parameters. Variables with p-value <0.1 in univariable models were then included in the multivariable model. The Levels of significance for multivariable model is P-value<0.05.

**Fig. S1. Flowchart of the study.**

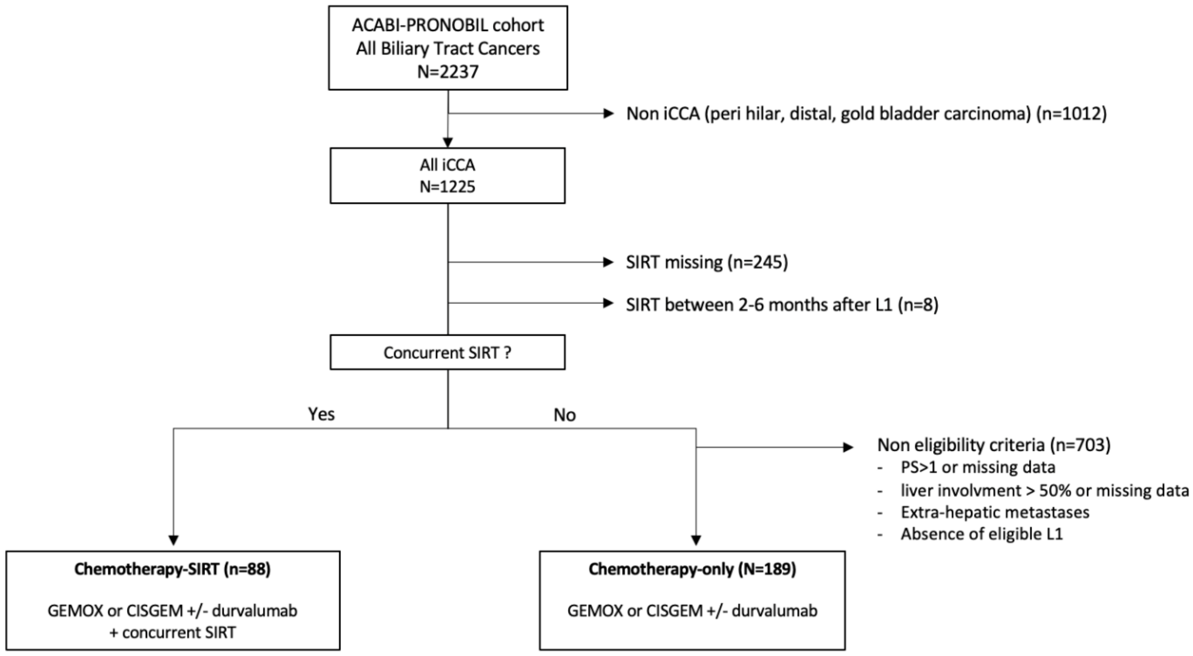


**Abbreviations**: CISGEM, cisplatin gemcitabine combination; ECOG PS, Eastern cooperative oncology group performance status; iCCA, intra-hepatic cholangiocarcinoma; GEMOX, gemcitabine oxaliplatin combination; SIRT, selective internal radiotherapy.

**Fig. S2. Caliper schema and matching curves**

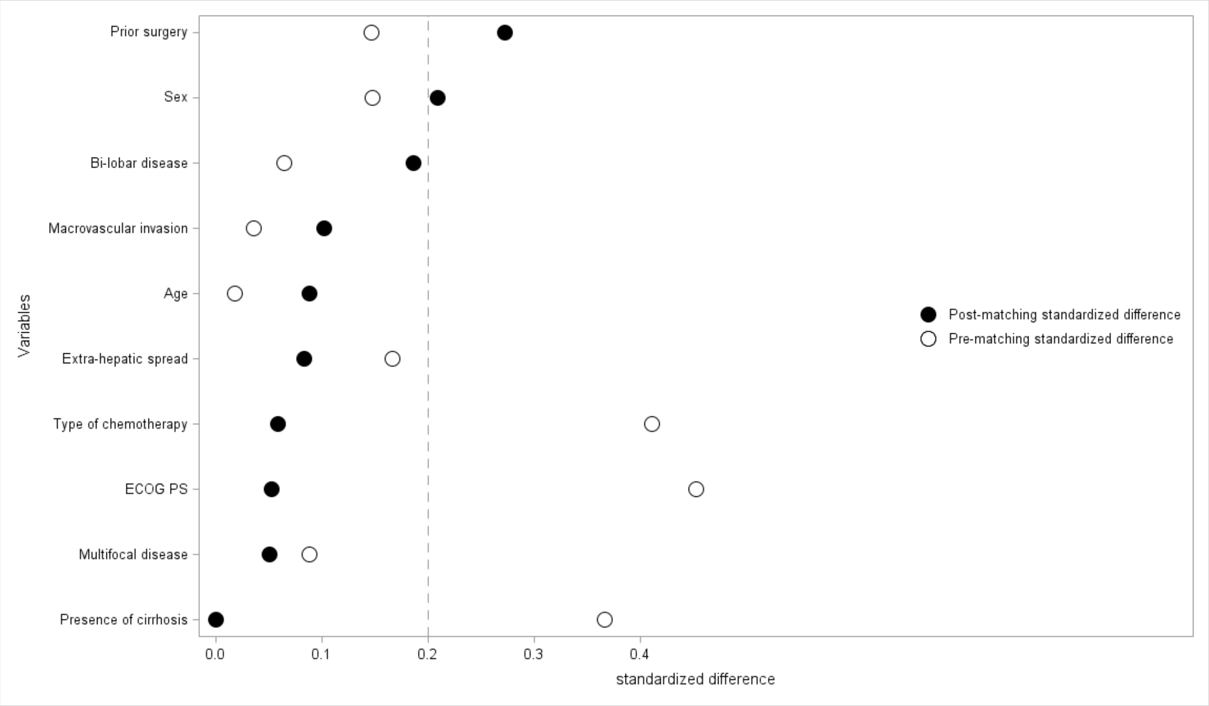


**Abbreviations**: ECOG PS: European cooperative oncology group performance status.
**Legend**: The standard differences compare baseline covariates between Chemo-SIRT group and Chemo alone group, a value close to 0 indicates groups with similar covariate distribution. Black circles are the standard differences in the overall population, white circles are the standard differences in the 1:1 matched population. After matching on propensity score, most variables have a lower standard difference, groups have similar characteristics.

**Fig. S3. Kaplan–Meier Curves of (A) progression-free survival and (B) overall survival in the matched 1:1 population.**

**A**

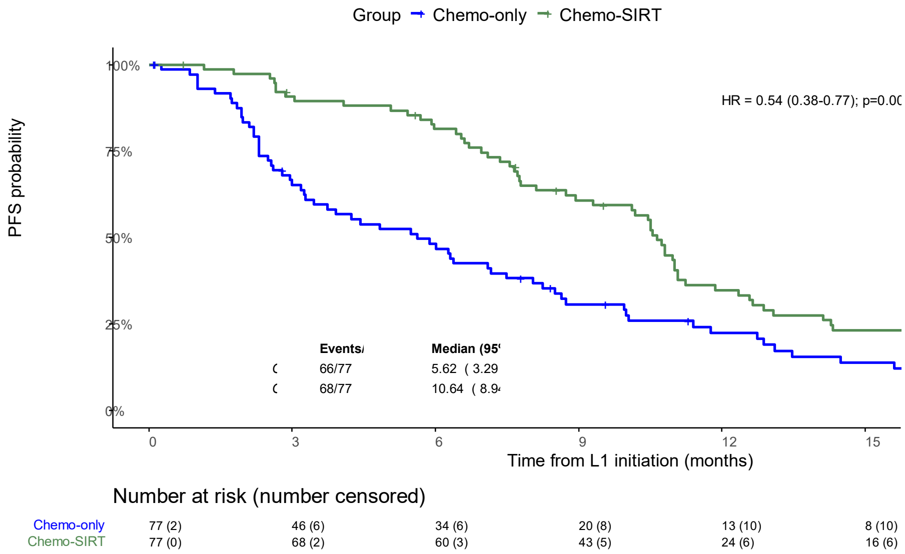


**B**

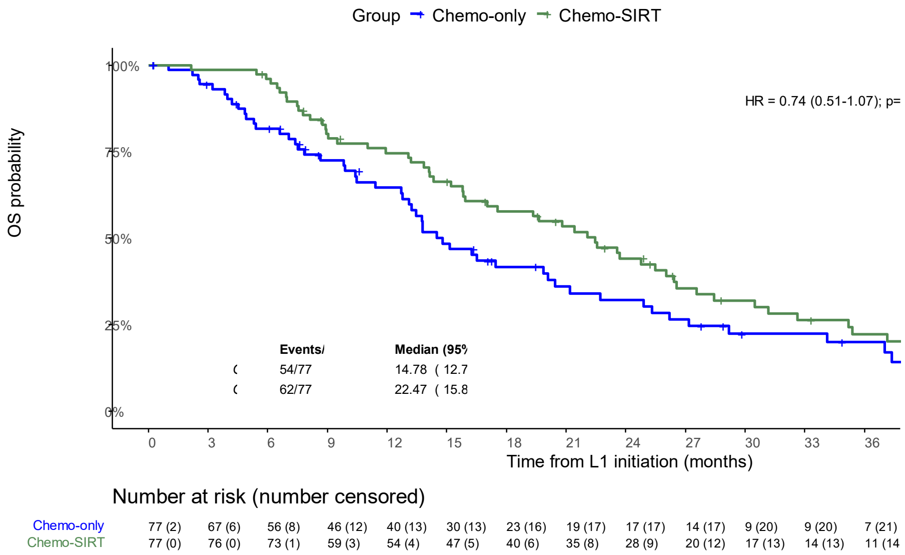


**Abbreviations:** SIRT, selective internal radiotherapy.
**Statistical analysis:** The matched 1:1 population was selected using a caliper of 0.15 based on the propensity score estimated with the multivariable logistic model. Survivals were estimated with the Kaplan-Meier method and hazard ratios and P-values with univariable Cox regression models. Levels of significance: $P<0.05$

**Fig. S4. Kaplan-Meier Curves of (A) progression-free survival and (B) overall survival in sensitivity analysis in early SIRT population.**

**A**

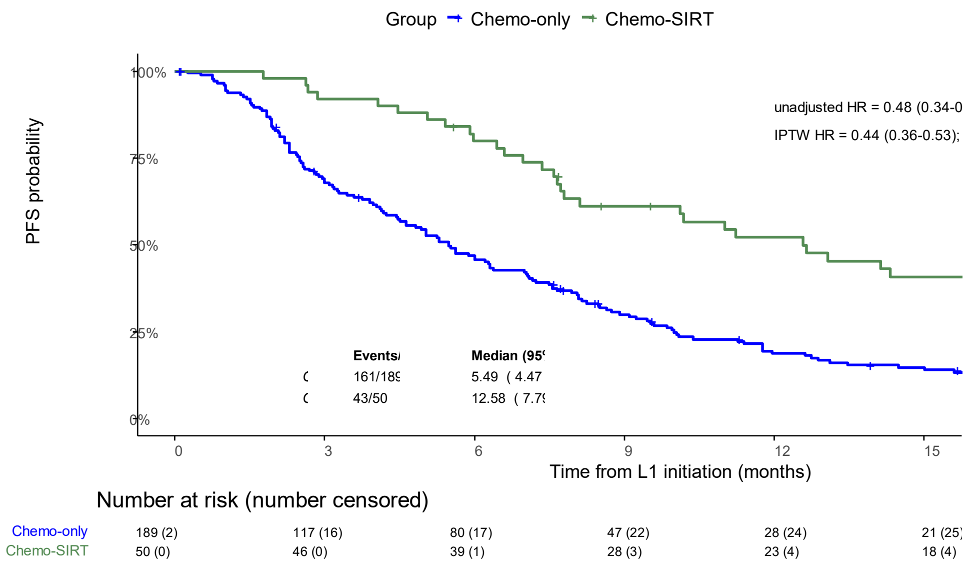


**B**

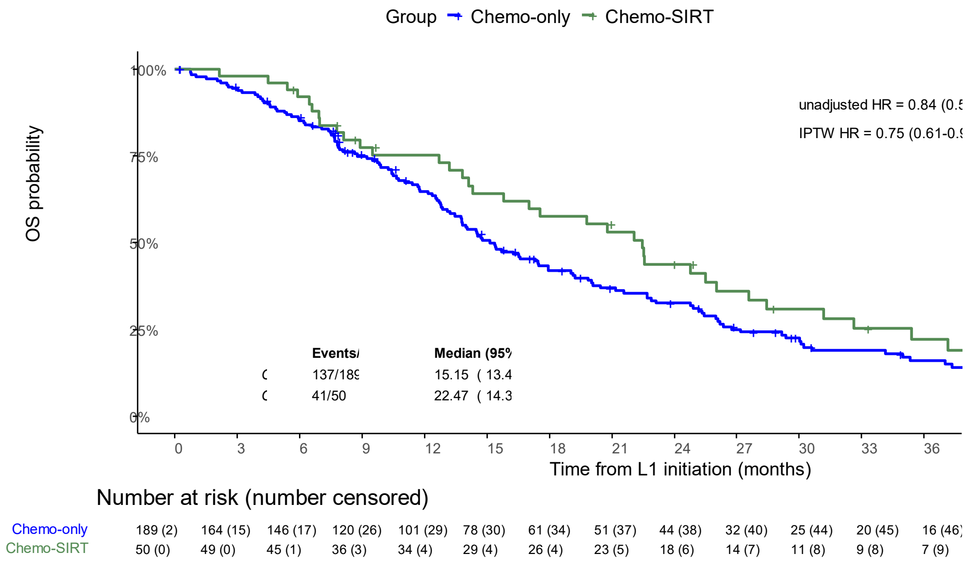


**Abbreviations:** SIRT, selective internal radiotherapy.
**Statistical analysis:** Levels of significance: P<0.05. Kaplan-Meier method to estimate the association between treatment group and PFS(A) and OS (B), described with medians and 95% confidence intervals (95% CI). Univariable Cox regression to assess hazard ratios (HR) with 95%CI for 1/unadjusted estimation and 2/corrected estimation with the inverse probability of treatment weighting method (IPTW) method. P-values are provided from Cox models.

**Fig. S5. Landmarks analysis of overall survival at 3 months (A) and 6 months (B).**

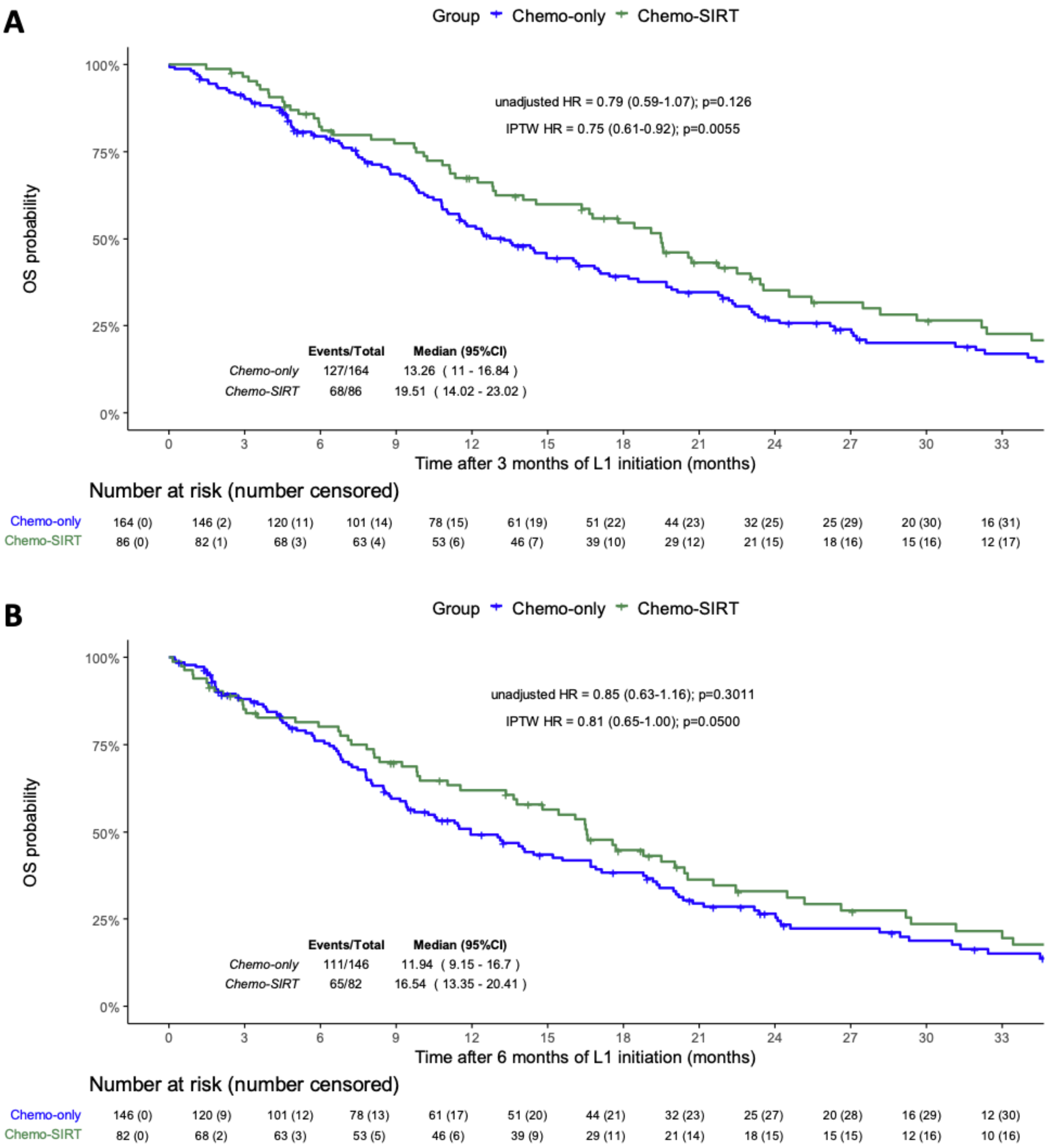


**Abbreviations:** SIRT, selective internal radiotherapy.
**Legend**: Patients still alive and followed up 3 months (A) or 6 months (B) after the initiation of the treatment were included in the analysis. In these analyses, OS started at 3 (A) and 6 (B) months after the initiation of the treatment.
**Statistical analysis:** Levels of significance: P<0.05. Kaplan-Meier method to estimate the association between treatment group and OS, described with medians and 95% confidence intervals (95%CI). Univariable Cox regression to assess hazard ratios (HR) with 95%CI for unadjusted estimation and corrected estimation with the inverse probability of treatment weighting method (IPTW) method. P-values are provided from Cox models.

**Fig. S6. Forest plots of overall survival by clinically relevant subgroups for chemotherapy-SIRT vs. chemotherapy only in the IPTW multivariate Cox regression model**

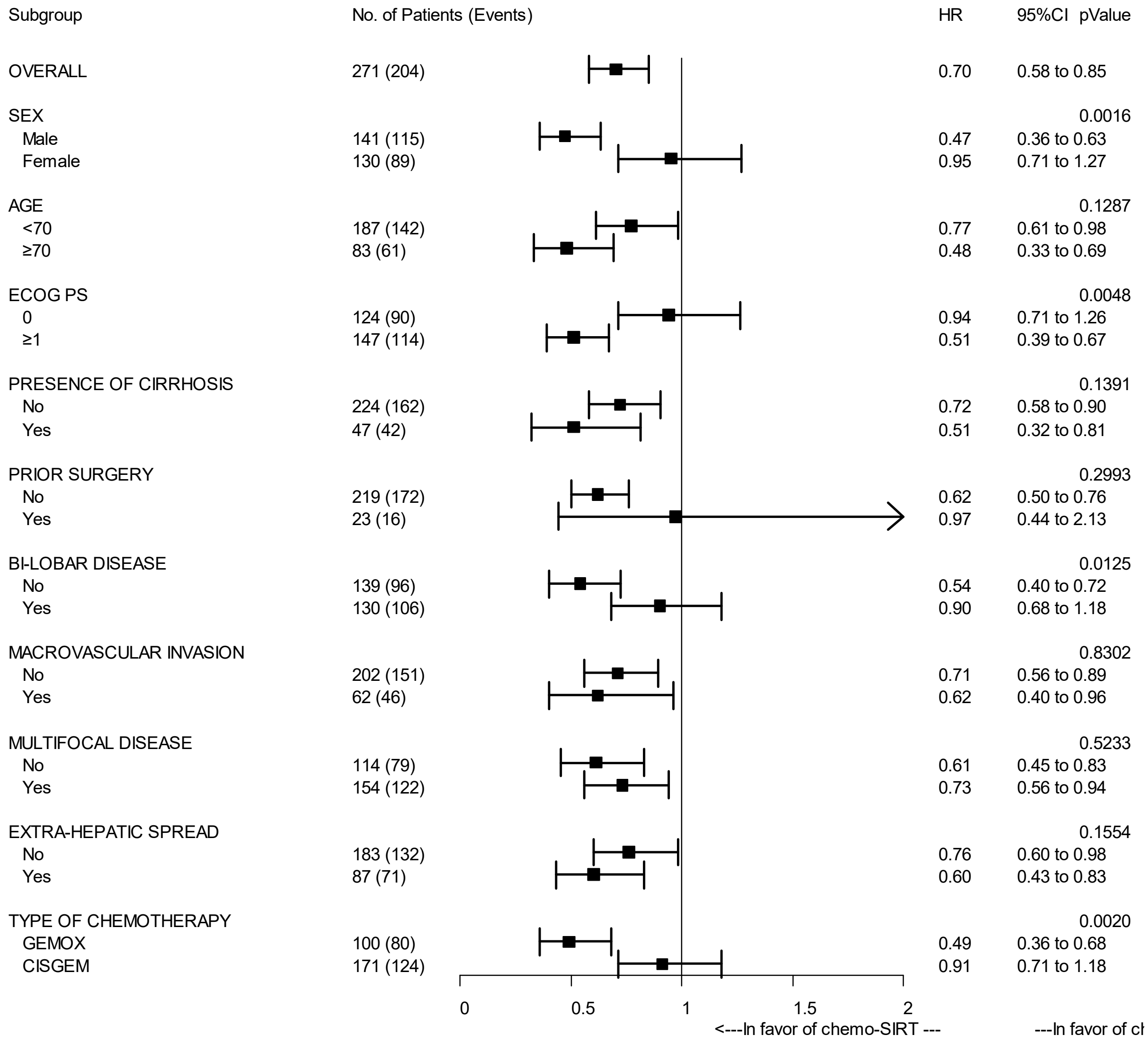


**Abbreviations**: CISGEM: gemcitabine - cisplatin; ECOG PS: eastern cooperative oncology group performance status; GEMOX: gemcitabine - oxaliplatin; SIRT: selective internal radiotherapy.

**Statistical analysis**: Levels of significance: p<0.1. Inverse probability of treatment weighting (IPTW) method applied in univariable Cox regression to assess the association between treatment group and PFS in each subgroup. The cox models include the treatment, the group and the interaction term between group and treatment. The p-values provided correspond to the interaction term p-value.

**Fig. S7: Histogram of treatment initiation year by treatment groups.**

**Statistical analysis:** Levels of significance: $p<0.05$. Wilcoxon Test.
