# Supplementary material for: First-line chemotherapy with selective internal radiation therapy for intrahepatic cholangiocarcinoma: The French ACABi GERCOR PRONOBIL cohort
Source: JHEP Rep. 2024 Nov 20;7(2):101279. doi: 10.1016/j.jhepr.2024.101279 (PMC11786833; doi:10.1016/j.jhepr.2024.101279)
Supplement: Multimedia component 2 [file mmc2.docx]

**JHEP Reports**

**CTAT methods**

Tables for a “Complete, Transparent, Accurate and Timely account” (CTAT) are now mandatory for all revised submissions. The aim is to enhance the reproducibility of methods.

- Only include the parts relevant to your study
- Refer to the CTAT in the main text as ‘Supplementary CTAT Table’
- Do not add subheadings
- Add as many rows as needed to include all information
- Only include one item per row

**If the CTAT form is not relevant to your study, please outline the reasons why:**

|  |
| --- |

- 1. **Antibodies**

| **Name** | **Citation** | **Supplier** | **Cat no.** | **Clone no.** |
| --- | --- | --- | --- | --- |
| Not applicable |  |  |  |  |

- 1. **Cell lines**

| **Name** | **Citation** | **Supplier** | **Cat no.** | **Passage no.** | **Authentication test method** |
| --- | --- | --- | --- | --- | --- |
| Not applicable |  |  |  |  |  |

- 1. **Organisms**

| **Name** | **Citation** | **Supplier** | **Strain** | **Sex** | **Age** | **Overall n number** |
| --- | --- | --- | --- | --- | --- | --- |
| Not applicable |  |  |  |  |  |  |

- 1. **Sequence based reagents**

| **Name** | **Sequence** | **Supplier** |
| --- | --- | --- |
| Not applicable |  |  |

- 1. **Biological samples**

| **Description** | **Source** | **Identifier** |
| --- | --- | --- |
| Not applicable |  |  |

- 1. **Deposited data**

| **Name of repository** | **Identifier** | **Link** |
| --- | --- | --- |
| Not applicable |  |  |

- 1. **Software**

| **Software name** | **Manufacturer** | **Version** |
| --- | --- | --- |
| Power point | Microsoft | 16.89.1 |
| SAS software | SAS Institute, Cary, NC | 9.3 |
| R software | SAS Institute, Cary, NC | 4.1 |

- 1. **Other (*e.g*. drugs, proteins, vectors etc.)**

| Not applicable |  |  |
| --- | --- | --- |
|  |  |  |

- 1. **Please provide the details of the corresponding methods author for the manuscript:**

| Gael S Roth, MD, PhD, Department of Hepato-Gastroenterology and Digestive Oncology; Team Immunology and Cancer - Institute for Advanced Biosciences, Grenoble–INSERM U1209 - CNRS UMR 5309, University Hospital of Grenoble-Alpes, Grenoble, France; Email: [groth@chu-grenoble.fr](mailto:groth@chu-grenoble.fr),  Tel: +33 4 76 76 51 68 |
| --- |

**2.0 Please confirm for randomised controlled trials all versions of the clinical protocol are included in the submission. These will be published online as supplementary information.**

| Not applicable |
| --- |
